# Management of open globe injury: a narrative review

**DOI:** 10.1038/s41433-024-03246-3

**Published:** 2024-07-31

**Authors:** Richard J. Blanch, David McMaster, Tim J. Patterson

**Affiliations:** 1 Institute of Inflammation and Ageing, University of Birmingham, Birmingham, UK; 2 Research and Clinical Innovation, Royal Centre for Defence Medicine, Birmingham, UK; 3 Department of Ophthalmology, University Hospitals Birmingham NHS Foundation Trust, Birmingham, UK; 4 Imperial College London, London, UK; 5 Northern Ireland Medical and Dental Training Agency, Belfast, UK

**Keywords:** Trauma, Eye diseases

## Abstract

Open globe injuries are a significant global cause of visual loss, including unilateral and bilateral blindness. Prognosis is predicted by injury severity, with lower presenting visual acuity and more posterior injuries associated with poor visual outcomes, although even the most severely injured eyes with no perception of light vision may regain some visual function. In addition to severity of the primary injury, the secondary injuries and complications causing poor outcomes include proliferative vitreoretinopathy (PVR) and endophthalmitis. Endophthalmitis is common after open globe injury, affecting up to 16.5% of patients. Systemic antibiotic prophylaxis is commonly used, with a limited evidence base, while intraocular antibiotics are less commonly used but have stronger supporting evidence of efficacy. Endophthalmitis rates are also reduced by prompt primary repair, which may also support recovery of visual acuity. PVR is not prevented or treated by any pharmacologic interventions in current clinical practice, but the incidence of post-traumatic PVR may be reduced by early vitrectomy within the first 4–7 days after injury. Ocular trauma training is often limited in Western ophthalmic surgical training programmes, and patients with ocular trauma often require the input of multiple subspecialists. In this context, it is important that patients have an overview and coordination of the different aspects of their care, with ownership by one lead clinician.

## Abstract (zh)

开放性眼外伤是全球范围内导致视力丧失的重要原因, 包括单眼和双眼失明。预后取决于损伤的严重程度, 后极部损伤较多以及较低的视力与视力不良有关, 尽管即使是无光感的最严重的眼外伤, 也可能恢复一些视觉功能。除原发性损伤的严重性外, 造成不良预后的继发性损伤和并发症包括增生性玻璃体视网膜病变(PVR)和眼内炎。

开放性眼外伤眼内炎很常见, 影响高达16.5%的患者。常用的全身性抗生素预防通常使用, 但证据基础有限, 而眼内抗生素使用较少, 却有更有力的证据支持其有效性。及时的一期修复也可降低眼内炎的发生率, 这也可能有助于视力的恢复。在目前的临床实践中, 任何药物干预都无法预防或治疗, 但创伤后4-7天内早期玻璃体切除可降低创伤后PVR的发生率。

西方眼科手术培训计划中, 眼外伤培训通常有限, 眼外伤患者通常需要多位专科医生的指导, 在这种情况下, 重要的是让患者全面了解和对其护理的不同方面, 并由一名主要临床医生负责。

## Introduction

Open globe injuries are a major cause of ocular morbidity, with a global estimated incidence between 3.5 and 4.5 per 100,000 [1, 2]. Open globe injuries account for the largest proportion of those having poor outcomes after ocular trauma, with visual outcomes variable and depending on comorbidities, injury severity, the effectiveness of management interventions and the occurrence of complications [3–5].

**Fig. 1:**
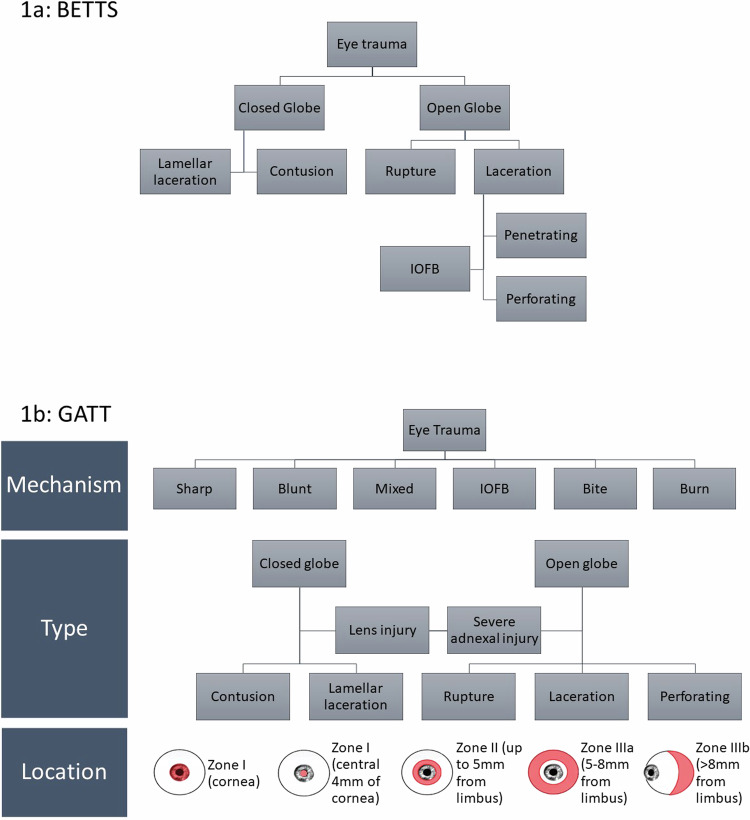
Ocular trauma classification systems. **a** Birmingham Eye Trauma Terminology System (BETTS), classifying injury type [6]. **b** International Globe and Adnexal Trauma Epidemiology Study (IGATES), classifying injury by mechanism, type and location [7]. More than one mechanism may apply: sharp injuries include low mass projectiles at high speed and sharp objects; blunt injuries include high mass projectiles at low speed; mixed mechanism includes very high energy injuries such as blast; IOFB, intraocular foreign body with entrance laceration; bite or sting e.g. from an animal; burn may be chemical or thermal. Type: closed globe injury has no full thickness wound of the sclera or cornea; contusion has no visible laceration; lamellar laceration has a partial thickness laceration; open globe injury has a full thickness wound of the sclera and/or cornea; a rupture is caused by a blunt object; a laceration is caused by a sharp object; a perforating injury has an entrance and an exit wound; lens injury and severe adnexal injury, including injury to the lacrimal drainage system, may occur in conjunction with open or closed injuries. Zone I includes the cornea and limbus.

Open globe injuries are any injuries involving full thickness defects of the eye wall and different classification systems are available. The most widely used system in published literature is the Birmingham Eye Trauma Terminology System (BETTS; Fig. 1a) [6], which was updated recently by the International Globe and Adnexal Trauma Epidemiology Study (IGATES) to incorporate additional features relevant to prognosis, including lid and lens injury and injury mechanism [7]. The classification systems reflect that open globe injuries may occur after blast injury, sharp or blunt trauma, and may be complicated by the presence of an intraocular foreign body (IOFB) and thermal or chemical burns. Sharp trauma is more likely to cause lacerating or penetrating injuries and blunt trauma more likely to cause ruptures. Injuries with an entry and an exit wound are perforating injuries, and along with ruptures, blast injuries and injuries associated with extensive lid trauma, tend to be those with the highest energy transfer to the eye and therefore the most severe injuries. Lens injury is a risk factor for endophthalmitis, and another marker of injury severity [8]. Zone of injury also affects prognosis and is defined by the international Ocular Trauma Classification Group as: Zone I, involving the cornea and limbus; Zone II, up to 5 mm posterior to the limbus; and Zone III, extending more than 5 mm posterior to the limbus [9]. The zone of injury therefore may denote the likelihood of retinal injury (Zone III) or of post-operative corneal scarring in the visual axis (central cornea). IGATES identified Zone IIIb (posterior to the equator) as having the worst prognosis [7].

A number of prognostic scoring systems have been identified to assess the severity of primary injury and its impact on prognosis, but none have yet improved on the Ocular Trauma Score (OTS), which gives primacy to presenting visual acuity [5, 9, 10]. A poor initial acuity therefore carries a poor prognosis for final visual outcome, especially when worse than counting fingers, although one study looking at the outcomes of eyes with no perception of light found a possible protective effect of iris injury, possibly relating to the disproportionate but reversible effect that hyphaema may have on visual acuity (Agrawal unpublished 2024).

The main complications causing secondary injury and poor anatomical and functional outcomes after open globe injury include expulsive haemorrhage, endophthalmitis, corneal scarring, proliferative vitreoretinopathy (PVR) and retinal detachment, leading to hypotony and phthisis, of which endophthalmitis and retinal detachment both feature in the OTS [5]. In addition to restoring anatomical structural integrity, avoidance of these complications, and timely detection and management when they do occur, should therefore be the main aim of management.

Whilst evidence for different aspects of open globe injury management exists, practice is variable, indicating a strong need for management guidelines, with responses to a recent survey of international ocular trauma centres indicating wide variation in all aspects of management [11]. With respect to antimicrobial prophylaxis, 76% administered pre-operative systemic antibiotic prophylaxis, 30% pre-operative topical antibiotics, and 55% intraoperative antibiotics. For timing of primary repair the urgency of surgical timing was also variable, with a three-way split between overnight primary repair, repair first case the next morning and repair whenever there was next routine space. For timing of vitrectomy there was also variation, with 21% considering the optimal timing within 4 days, 18% within 4–7 days and 46% ≥7days after primary repair [11].

## Medication

Endophthalmitis is a blinding intraocular infection, that is usually caused by Gram-positive cocci but may be caused by a wide variety of bacteria and fungi [12]. Endophthalmitis is relatively common after open globe injury, with reported incidence rates are between 0% and 16.5% [13]. Antibiotic prophylaxis is therefore commonly prescribed, although there is no consensus on administration, route, or choice of medication [11, 14]. Prophylaxis may be pre-operative, and systemic or topical, allowing prompt administration as soon as the diagnosis is made, or suspected, by ophthalmologists or non-specialists, or may be intra-operative, which adds intraocular administration as a potential route.

A recent systematic review found that whilst there was no prospective evidence that pre-operative antibiotic prophylaxis reduced endophthalmitis risk, the single included prospective study was consistent with no benefit up to an absolute risk reduction of 6.1% [14]. The included study also looked at topical antibiotic prophylaxis, finding no evidence of risk reduction. As the potential consequences for the patient of developing endophthalmitis, in terms of loss of vision and loss of the eye, are high and the risks of single doses or short courses of oral antibiotics are relatively low, antibiotic prophylaxis remains in common usage with decisions weighted on a case-by-case risk:benefit assessment [11]. When antibiotic prophylaxis is used, one review found moderate evidence that oral ciprofloxacin was non-inferior to intravenous antimicrobials (Ceftazidime, Cefazolin and Vancomycin were used in included studies), consistent with the high bioavailability of oral fluoroquinolones [14, 15].

In contrast to pre-operative oral antibiotic prophylaxis, which is widely used with little supportive evidence, intra-operative antibiotic prophylaxis has strong evidence of efficacy but is less commonly administered [11, 16, 17]. A 2017 meta-analysis included data from three randomised controlled trials of intraocular (intravitreal or intracameral) antibiotic injection at the time of primary repair, finding that intravitreal injection of gentamicin reduced the endophthalmitis rate from 6% to 0% [16]. Whilst intravitreal gentamicin may raise valid safety concerns, and it is necessary to ensure that the retina is flat before performing intravitreal injection, intraocular antibiotic prophylaxis is routine after cataract surgery, when endophthalmitis is much less common, and the argument for intraocular prophylaxis at completion of primary repair is therefore compelling.

## Primary repair

In the UK National Health Service (NHS), primary repair is not usually conducted overnight, but may be conducted in the evening, with the usual standard being that cases should be repaired within 12–24 h [18]. This commonly means that primary repair is performed on an elective list the day after presentation, displacing previously scheduled cases. In other countries (such as the USA) primary repair may be performed as an emergency procedure after presentation, with surgery performed overnight where necessary [11].

Significant data from outside of ophthalmology associates “after-hours” (not defined) surgery with worse mortality and morbidity outcomes, outlined in a recent cross-specialty systematic review, and meaning that the necessity for after-hours operating must be justified [19]. Equally, the decision by British Armed Forces not to deploy ophthalmology to Afghanistan was associated with delayed treatment and worse open globe injury outcomes with higher endophthalmitis rates than other military healthcare systems that practiced prompt primary repair, despite systemic antibiotic prophylaxis [20, 21].

Appropriately timed primary repair after injury is necessary to minimise the risk of complications such as endophthalmitis and expulsive haemorrhage [11, 22]. Retrospective data indicates that delays to primary repair beyond 24 h increase the risk of endophthalmitis and are associated with a reduction in visual outcomes [8, 22–28]. However, other studies did not find an effect of time to primary repair (within 24 h) on final visual acuity [29, 30]. Given the variability in injury severity (a major determinant of outcome), and the fact that in most of the world, primary repair is performed within 24 h or shortly thereafter, it is unsurprising that retrospective studies would not always detect an effect of primary repair timing on outcome [11, 24, 31, 32]. However, the effect of delay on endophthalmitis seems to be consistent and not abrogated by the administration of systemic antibiotic prophylaxis [21].

Most studies investigating the effect of primary repair timing have selected a cut-off of more than, or less than 24 h; however, one paper found a detrimental effect of delay beyond 12 h, with a greater occurrence both of endophthalmitis and others have found a greater rate of return to theatre for wound leak [26, 33]. If an effect exists, it is likely that 24 h is an arbitrary period of time, and that any detrimental effect increases continuously with greater delay, so that repair at 12 h is preferable to 24 and there is little difference between 23 and 25 h. Thus, all other things being equal, repair as soon as possible seems advisable, but that must be weighed against the risks and costs of operating out of hours and the availability of staff and equipment. Nonetheless, given the retrospective data and standard practice around the world, it is difficult to defend policies that plan for primary repair more than 24 h after injury.

## Vitrectomy

Proliferative vitreoretinopathy (PVR) is an intraocular scarring process characterised by the growth and contraction of cellular membranes within the vitreous cavity, and on both sides of the retinal surface as well as intraretinal fibrosis and is usually precipitated by retinal breaks allowing RPE cells into the vitreous [34]. After open globe injury, rhegmatogenous retinal detachment (RRD) occurs in 29% (26%-32% CI) of patients within the first month and up to 43% of eyes ultimately develop blinding PVR with total retinal detachment without vitreoretinal intervention, and up to 50% develop PVR [35, 36].

There are no pharmacological interventions in current practice that prevent or modulate the occurrence of PVR, and the only effective treatment is vitrectomy. Current practice in terms of timing of vitrectomy after open globe injury is very variable, with no consensus on which patients require vitrectomy, or how quickly it should be performed [11]. Retrospective observational studies suggest that PVR rates are lower with vitrectomy performed within 4–7 days of injury [37, 38], and limited randomised control trial data support this [39]. The risk of RRD and PVR are highest when the retina is involved in the injury, and such injuries are described as Zone III (Fig. 1b).

In addition to prevention and treatment of PVR, some patients require vitrectomy to remove intraocular foreign bodies (IOFB). US military data from the conflict in Iraq describe management of IOFB with prompt primary repair, but with vitrectomy delayed for up to 120 days, with no cases of endophthalmitis, although they did report a PVR rate of 21% [20]. It therefore seems likely that vitrectomy after Zone III injuries, especially those with RRD or vitreous haemorrhage obscuring the fundal view, should be performed within 4–7 days. It is less clear however, which other injuries (if any) require vitrectomy for PVR prevention or how rapidly IOFB should be removed when the retina is intact.

## Eye removal

In all prognostic scoring systems, eyes with no perception of light (NPL) after open globe injury have a poor prognosis [5, 40]. One of the most feared complications of open globe injury is sympathetic ophthalmia (SO), a granulomatous autoimmune uveitis which occurs at a rate of 0.06-0.19% and may cause visual loss in the fellow eye [41, 42]. Historic teaching was that severely injured eyes should be removed within two weeks of injury to prevent SO, but neither the timescale nor the efficacy of such intervention is evidence-based.

Eyes with visual acuity of NPL have reported rates of visual recovery (to better than NPL) in up to 89% in papers specifically focussed on the outcomes of eyes with NPL vision [43]. The rate of recovery to better than NPL of OTS I eyes (e.g. NPL vision with RAPD after rupture) was 26%, and 73% in OTS II eyes (e.g. NPL with RAPD after penetrating injury) in the original description of the ocular trauma score, suggesting that, whilst there may be an element of positive publication bias in series specifically focussed on the outcome of eyes with NPL vision after trauma, some recovery is common. In addition, the determination of NPL visual acuity in patients immediately after trauma may be less reliable than at other times, meaning that significant visual recovery in patients presenting with NPL vision cannot be ruled out.

It is true that SO is rarely reported after primary eye removal, but a recent meta-analysis of retrospective studies comparing the risk of SO with different management strategies found that the rate of SO after primary repair was 0.15% (95% CI 0.00% to 0.33%), compared to 0.00% - 0.21% after eye removal, with a 95% confidence interval of the absolute risk reduction of 0.11% (in favour of primary repair) to -0.31% (in favour of eye removal) [44]. Therefore, there was no evidence that eye removal prevents SO, but the confidence intervals indicated that any potential effect would be small, with at least 323 eyes needing to be removed to prevent one case. There is also no evidence to support enucleation over evisceration (which has a better cosmetic result). One study examined the effect of not repairing an injured eye and found 2/109 (2%) patients developed SO after non-surgical open globe injury management – a higher rate than after primary repair or eye removal [45]. The existing evidence therefore suggests that eyes should be repaired where possible.

## Ocular traumatology

Patients suffering open globe injuries all require primary repair and at least half require vitrectomy. In addition, many will also require oculoplastic input, corneal and glaucoma surgery, and a smaller proportion will need to see a uveitis specialist, and patients may therefore see multiple subspecialists. In Western civilian healthcare systems, ocular trauma is uncommon and the UK Ophthalmology Curriculum specifies a minimum of one open globe repair before completion of training and the Accreditation Council for Graduate Medical Education (ACGME) in the USA specifies a minimum of four [46, 47]. In survey data, most US residents had performed fewer than six globe repairs (maximum <50), and UK trainees an average of two (maximum six), meaning that the acquisition of ocular trauma knowledge, skills and experience (KSE) may be limited in US and UK training programmes, and ocular trauma fellowships are not commonly offered (although emergency eye care fellowships are), so there are few routes to gaining this experience for a trainee ophthalmologist [48, 49].

As in major trauma management, management of ophthalmic trauma therefore requires a team effort, with good multidisciplinary and multi-professional communication. The team have different skill sets and no single clinician has the knowledge to manage all aspects of the patients’ care, but the lead clinician needs to coordinate and balance between the subspecialists and provide an overview of the patient’s care [50]. Given the potentially limited KSE in open globe injury management in individual subspecialists, this coordination and overview of the care of the ocular trauma patient is especially needed in our subspecialised healthcare systems.

## Conclusions

Prompt primary repair, combined with intraocular administration of antibiotics, minimises endophthalmitis risk and may optimise visual outcome. Optimal timing is based on retrospective studies, with 24 h being the most commonly used arbitrary cut-off, and it is likely that sooner is better. There is no evidence to support eye removal to prevent sympathetic ophthalmia when primary repair is possible, even in the most severely injured eyes.
